# Barriers and enablers of weight management after breast cancer: a thematic analysis of free text survey responses using the COM-B model

**DOI:** 10.1186/s12889-022-13980-6

**Published:** 2022-08-20

**Authors:** Carolyn Ee, Freya MacMillan, John Boyages, Kate McBride

**Affiliations:** 1 NICM Health Research Institute, Western Sydney University, Locked Bag 1797, Penrith, NSW 2751 Australia; 2 Translational Health Research Institute, Western Sydney University, Penrith, NSW Australia; 3 School of Health Sciences, Western Sydney University, Penrith, NSW Australia; 4 ICON Cancer Centre, Sydney Adventist Hospital, Wahroonga, NSW Australia; 5 Faculty of Health and ANU College of Health & Medicine, Australian National University, Canberra (Australian Capital Territory), Australia; 6 School of Medicine, Western Sydney University, Penrith, NSW Australia

**Keywords:** Breast cancer, Weight, Physical activity, Qualitative, Supportive care, COM-B

## Abstract

**Background:**

Weight gain is common after breast cancer. The aim of this study was to identify and describe the barriers to and enablers of successful weight management for women with breast cancer.

**Methods:**

This was a combined inductive and deductive framework analysis of free text responses to an anonymous cross-sectional survey on weight after breast cancer. Women were recruited mainly through the Breast Cancer Network Australia Review and Survey Group. We applied deductive thematic analysis to free text responses to questions on barriers, enablers, research priorities, and one open-ended question at the end of the survey using the Capability, Opportunity, Motivation and Behaviour (COM-B) model as a framework. Subthemes that arose from the inductive analysis were mapped onto the COM-B model framework. Findings were used to identify behaviour change intervention functions.

**Results:**

One hundred thirty-three women provided free text responses. Most women were of Caucasian origin and had been diagnosed with non-metastatic breast cancer, with a mean age of 59.1 years. Women's physical capability to adopt and sustain healthy lifestyle habits was significantly affected by treatment effects and physical illness, and some lacked psychological capability to self-regulate the face of stress and other triggers. Limited time and finances, and the social impact of undergoing cancer treatment affected the ability to control their diet. Frustration and futility around weight management were prominent. However, some women were confident in their abilities to self-regulate and self-monitor lifestyle behaviours, described support from friends and health professionals as enablers, and welcomed the physical and psychological benefits of being active in the context of embracing transformation and self-care after cancer.

**Conclusion:**

Women need specific advice and support from peers, friends and families and health professionals. There is a substantial gap in provision of supportive care to enable women to adopt and sustain healthy lifestyles. Environmental restructuring (including financial support), incentivization (creating an expectation of looking and feeling better), persuasion and coercion (aiming to prevent recurrence), and equipping women with specific knowledge and skills, would also facilitate optimal lifestyle behaviours and weight management.

**Supplementary Information:**

The online version contains supplementary material available at 10.1186/s12889-022-13980-6.

## Background

The most common cancer amongst women is breast cancer [1, 2] with the global incidence predicted to rise from 2 million new cases in 2018 to 3 million in 2040 [1]. Particularly for post-menopausal women, obesity or being overweight is a well-known risk factor [3]. Obesity at diagnosis and weight gain after treatment has been linked to higher recurrence, breast cancer mortality and all-cause mortality rates [2, 4, 5]. Weight gain is a common occurrence after the diagnosis of breast cancer and has been linked to lower quality of life [2]. Factors responsible for this weight gain include the use of chemotherapy, younger age at diagnosis, induced menopause, and reduction in physical activity [2, 6]. Effective weight loss interventions are typically multimodal, incorporating diet, exercise and psychosocial support [7] however women with breast cancer have described multiple barriers to successful adoption and maintenance of weight management strategies [8].

Given the growing population of breast cancer survivors and the link between weight gain and adverse health outcomes, research into weight management after breast cancer is of critical importance. An understanding of the barriers and enablers of successful weight management after breast cancer is needed in order to inform the development of appropriate interventions.

However, quantitative assessment lacks the richness and depth of qualitative evaluation and does not adequately capture the experience of weight management after breast cancer. Qualitative research seeks to understand the experiences and meaning in participants’ lives and can result in a deeper and more nuanced and comprehensive understanding of illness or behavior than quantitative research. The aim of this study was to identify and describe the barriers and enablers of successful weight management in women with breast cancer, using thematic analysis of 250 free text responses to our survey on weight management after breast cancer in women living in Australia [9]. We used a theory-based approach to our analysis in order to fully understand the context in which weight loss behaviours (restricting diet and increasing physical activity) occur in our sample, by using the Capability Opportunity Motivation – Behaviour (COM-B) theoretical model proposed by Michie *et.al.* [10]. The COM-B model was developed after a comprehensive review of nineteen behaviour change frameworks and proposes that there are three essential components to any behaviour: capability (having the knowledge, skills and abilities to engage in a particular behaviour); opportunity (external factors that make a behaviour possible); and motivation (internal processes that influence decision making and behaviours). These form the hub of a “behaviour change wheel” around which are placed nine intervention functions that are aimed at addressing any gaps in capability, opportunity and motivation. Understanding behaviour within the framework of the COM-B therefore provides a foundation on which to select intervention strategies that can bring about behaviour change.

## Methods

### Study design and inclusion criteria

We conducted a cross-sectional, self-administered, anonymous survey using the online survey program Qualtrics [11] between November 2017 and March 2018. Ethics approval was granted by the Western Sydney University Human Research Ethics Committee (H12444, October 2017). Our methods have been previously described [9]. Briefly, we recruited women mainly through the Breast Cancer Network Australia (BCNA) Review and Survey Group. BCNA is the largest breast cancer advocacy group in Australia. Limiting research at BCNA to the Review and Survey group allows researchers to access women who are engaged in the research process, while protecting the rest of BCNA from frequent research requests. Women were also recruited through online breast cancer support groups and women’s health organisation social media pages in Australia. Any woman living in Australia who self-identified as having a breast cancer diagnosis was eligible to complete the survey. Participants were informed that the aim of the survey was to explore weight change after breast cancer. Participants were provided with an electronic copy of the Participant Information Sheet via a weblink on the survey website prior to commencing the survey, and were informed that consent was implied upon commencement.

### Data analysis

Details of the survey instrument have been previously described [9]. We conducted thematic analysis of free text responses to the questions outlined in Table 1. Three questions were multiple choice questions about barriers and enablers of successful weight loss and weight maintenance and research priorities for addressing weight concerns after breast cancer, and included a long free text option. The fourth question was a free text question that asked if survey participants had anything they would like to add. The thematic analysis approach was selected as it suits questions related to people’s experiences, views or perceptions, and is a commonly used method for identifying, reporting and interpreting patterns within qualitative data [12].

**Table 1:**
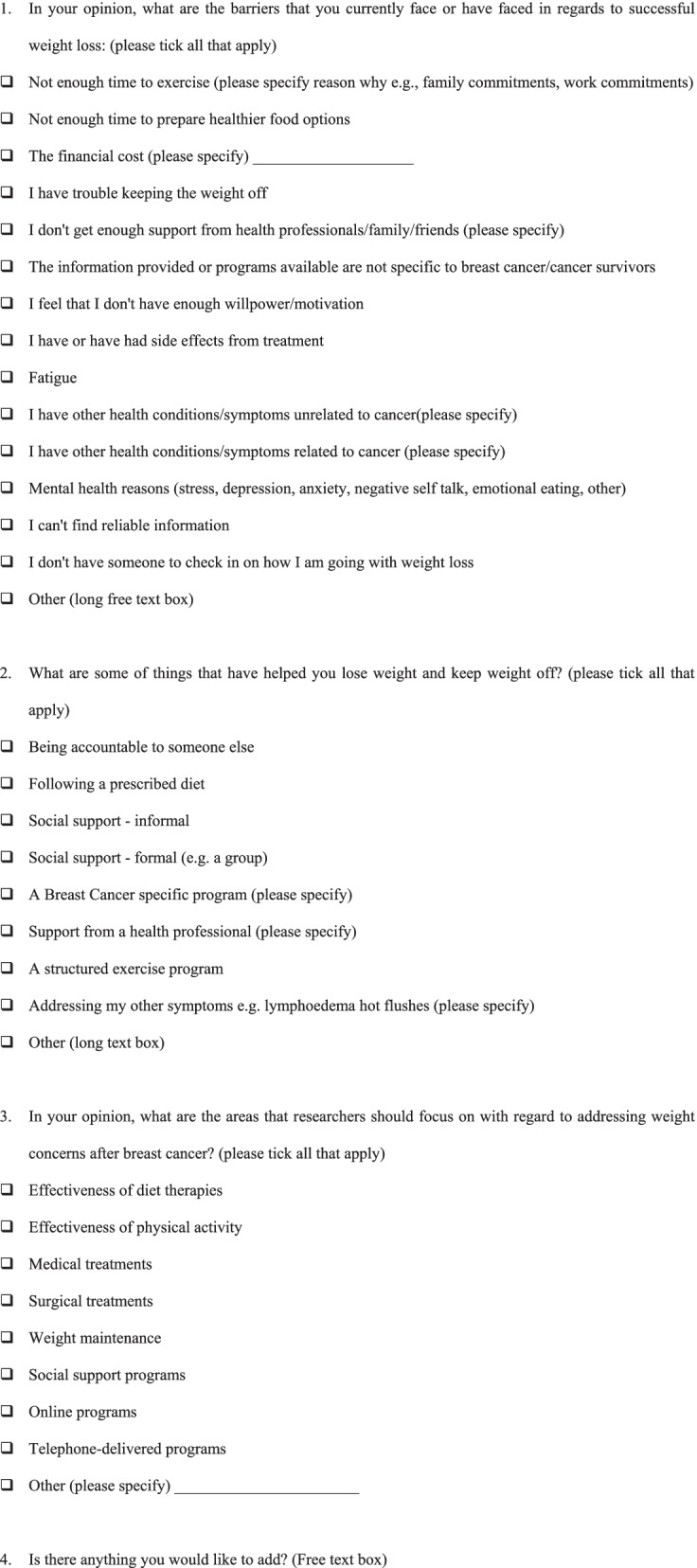
Survey questions containing free text options included in this study

Free text responses were retrieved from the online survey data and imported into Excel v16.55 software. Three researchers (KM, FM, CE) familiarised themselves with the open text responses. We used a framework analysis method [13], with participants as rows and themes and subthemes as columns. Two frameworks were developed, one for barriers and one for enablers of weight loss and weight loss behaviours. This method of analysis allowed us to identify consistent patterns and relationships within and across themes. The framework was developed both inductively (researcher-driven – KM and FM) [14] and deductively (using the COM-B model – CE and KM). The behaviours identified for the purposes of this study were controlling caloric intake and participating in physical activity. Where it was unclear which behaviour women were referring to in the free text responses, we coded the responses as “both [behaviours] or unclear”. Each text response was first coded to whether it was a barrier or an enabler, then whether the participant was referring to restricting diet, increasing physical activity, or whether the text response referred to both or was unclear. The response was then coded using the COM-B model into the broad components of Capability, Opportunity or Motivation, and further coded into sub-components of Physical or Psychological Capability, Physical or Social Opportunity, and Reflective or Automatic Motivation. Last, the response was assigned a subtheme that arose from the inductive coding. Continued revision of the categories and emerging themes took place with the researchers searching for sub-topics and new insights into each category. The researcher used strategies such as independent coding, use of excerpts to support statements, and consensus meetings throughout to ensure that study inter-rater reliability and rigour were upheld by ensuring trustworthiness in coding.

Once this stage of coding was complete, we mapped the codes to intervention functions as described in the COM-B matrix by Michie *et.al.* Michie and colleagues identified nine intervention functions based on a comprehensive review of 19 behaviour change frameworks, each mapping on to one or more components of the COM-B model [10].

## Results

A total of 309 women responded to the survey, of which 133 included 250 free text responses relevant to this study (Table 2). Most women were of Caucasian origin (94.1%, *n* = 144) with a mean age of 59.1 years (SD = 9.1, range 37–78, *n* = 128). Most women who had provided a free text response had been diagnosed with non-metastatic breast cancer (92.5%, 123/133) with an average of 7.9 years since diagnosis (SD 5.1, range 1–32 years, *n* = 130). The mean age at diagnosis was 51.3 years. Mean current BMI was 27.89 kg/m^2^ (SD 6.1). Mean weight gain was 4.6 kg (SD 9.98, *n* = 127) and 55.1% (70/127) of this sample reported they had gained more than 5% of their body weight at diagnosis. Other details of diagnosis and treatment are provided in Table 3.

**Table 2:** Demographic characteristics of survey respondents who provided a free text response

	Description	N (responses)	%
**State (*n* = 133)**			
	Australian Capital Territory	6	4.5
	New South Wales	38	28.6
	Victoria	37	27.8
	Queensland	18	13.5
	Western Australia	16	12.0
	South Australia	28	13.5
**Education (*n* = 133)**			
	High school- year 10	8	6.0
	High school- year 12	13	9.8
	Vocational College	27	20.3
	Bachelor’s degree	36	27.1
	Postgraduate degree	49	36.8
**Ethnicity (*n* = 132)**			
	European/Anglo Saxon/Caucasian	125	94.0
	Other (Oceanic, Asian, Indian, South/Central American, Mixed Ethnicity)	7	5.2
	Missing	1	0.8
**Employment (*n* = 132)**			
	Employee	54	40.6
	Self-employed	16	12.0
	Home duties/caring for children or family	7	5.3
	In education (going to school, university, etc.)	1	0.75
	Doing voluntary work	6	4.5
	Unable to work because of illness	3	2.3
	Retired	45	33.8
	Missing	1	0.75
**Relationship Status (*n* = 133)**			
	Single	17	12.8
	Married/de facto (living with partner)	100	75.2
	In a relationship (not living with partner)	4	3.0
	Divorced/separated	8	6.0
	Widowed	4	3.0
**Weight gain pattern (*n* = 132)**			
	Gained weight overall	80	60.2
	Lost weight overall	18	13.5
	Weight remained stable	20	15.0
	Weight fluctuated a great deal	14	10.5
	Missing	1	0.8

**Table 3:** Diagnoses and treatments received

	Description	N	%	Missing n (%)
**Diagnoses**				-
	Ductal Carcinoma In Situ (DCIS)	15	11.3	
	Localized breast cancer	108	81.2	
	Metastatic breast cancer	5	3.8	
	Inflammatory breast cancer	< 5	0.8	
	Other including second primary	< 5	3.0%	
**Treatment to the Breast**				1 (0.8%)
	Lumpectomy alone	< 5	0.7%	
	Lumpectomy and radiation	57	42.9%	
	Mastectomy alone	28	21.1%	
	Mastectomy and radiation	35	26.3%	
	Lumpectomy and mastectomy alone	< 5	2.3%	
	Lumpectomy, mastectomy and radiation	7	5.3%	
	Double mastectomy	< 5	0.8%	
**Reconstruction after mastectomy (*n* = 73)**				-
	No	40	54.8%	
	Immediate	16	21.9%	
	Delayed	17	23.3%	
**Treatment to the Axilla (*n* = 71)**				< 5 (5.6%)
	Sentinel node biopsy only	7	9.9%	
	Axillary dissection ± Sentinel node biopsy	25	35.2%	
	Axillary dissection ± Sentinel node biopsy + radiation	34	47.9%	
	Radiation only	< 5	1.4%	
**Intravenous Systemic Therapy**				
	Chemotherapy without Herceptin	73	54.9%	
	Herceptin only	< 5	1.5%	
	Chemotherapy + Herceptin	24	18.0%	
	None/not reported	34	25.6%	
**Hormonal Treatments**				
	Tamoxifen alone	25	18.8%	
	Aromatase inhibitors alone	18	13.5%	
	Other/combination	< 5	3.0%	
	None/not reported	86	64.7%	
**Current use of hormone therapy**				
	Yes	52	39.1%	

### Findings from the thematic analysis

Themes and subthemes are described in Table 4 and additional quotes can be found in Additional Tables 1, 2, 3, 4, 5 and 6. Participants are identified by a unique ID number.

**Table 4:** Themes and subthemes

COM-B component	Barrier	Enabler
**Capability – attribute of a person makes a behaviour possible or facilitates it (together with opportunity); capacity to carry out a behaviour**		
***Physical –**** capability that involves a person’s physical function, skill, strength or stamina*	*Diet* Limited food options due to other health conditions Symptoms from treatment affects eating habits *Exercise* Physical illness makes exercise difficult (both cancer and non-cancer related) *Both/unclear* Menopause, physical illness and endocrine therapy makes weight loss difficult	*Exercise* Given specific exercises to use by a trained professional
***Psychological**** – Knowledge or psychological skills, strength or stamina to engage in the necessary mental processes*	*Diet* Lack of interest/vague advice from health professionals Unable to regulate eating in response to reasons apart from hunger “Self sabotage” *Both/unclear* Distress Lack of information	*Diet* Self-regulation Specific information about diet (including doing own research) *Exercise* Creating good habits *Both/unclear* Specific program and support Having a clear goal Psychological support, positive mindset Self-regulation and monitoring Self-efficacy
**Opportunity** – **attribute of an environmental system that makes a behaviour possible or facilitates it, together with capability**		
***Physical**** – Opportunity afforded by the environment, including time, resources, locations, cues, physical “affordance”*	*Diet* Availability of high calorie foods *Exercise* Environment (heat) *Exercise and both/unclear* Lack of time due to study/work/family commitments, general overwhelm Financial cost	*Diet* Limiting access to high calorie foods *Exercise* Having a dog to walk (ID 283) *Both* Affordable programs
***Social –**** Opportunity afforded by interpersonal influences, social cues and cultural norms that influence the way that we think about things*	*Diet* Other people cooking/social eating *Unclear* Lack of support from friends/health professionals Medical advice/social pressure to not lose too much weight	*Exercise* Peer support or support from family/friends *Both/unclear* Feeling normal again Individualised approach
**Motivation – a mental process that energises and directs behaviour**		
***Reflective**** – involves plans (self-conscious intentions) and evaluations (beliefs about what is good or bad)*	*Diet* Enjoyment (or dislike) of food and cooking *Both/unclear* Beliefs that she cannot lose the weight Frustration at not being able to lose weight	*Exercise* Financial and other incentives, fun and welcoming environment Helps mind and body Look better, feel better *Both/unclear* Knowing the cause of weight gain Wanting to avoid recurrence Wanting to get fitter Told to lose weight by someone she trusts Cancer is a wake-up call
***Automatic**** – involves emotional reactions, desires (wants and needs), impulses, inhibitions, drive states and reflex responses*	*Diet:* Eating/drinking for reasons apart from hunger *Exercise:* Dislikes exercise *Both/unclear:* Fear of recurrence	*Both/unclear* Doesn’t like the feeling of being overweight

### Capability – physical

#### Barriers

By far the most prominent barrier faced by women in our study related to physical capability to exercise or control diet. These were both cancer-related (including treatment side effects) and caused by a wide range of non-cancer illnesses. For some, eating habits were affected by existing illness or symptoms from treatment. Many women attributed being menopausal and being on endocrine therapy as a cause for difficulty in maintaining a healthy weight. Women on endocrine therapy expressed frustration at not seeing results even with great effort, and at not being warned about the possibility of weight gain with endocrine therapy.

“Even though I no longer use it [tamoxifen], I still cannot lose weight unless I eat hardly anything” (ID 109)

Women reported often feeling fatigued and experienced a wide range of other health problems like hypothyroidism and arthritis, which restricted their ability to maintain physical activity. Some women experienced aromatase-inhibitor induced arthralgia, which also impacted on their ability to exercise especially if it exacerbated existing arthritis or musculoskeletal problems.

Chemotherapy-induced peripheral neuropathy, as well as the interaction of chemotherapy with other medications already being taken for existing chronic conditions such as arthritis, also hindered being able to exercise. Ongoing effects from other treatment modalities were also reported to act as a barrier to exercise. For example, some participants described how their lung and cardiac function had been potentially affected by radiotherapy which in turn reduced their exercise capacity. Surgery also left its mark in other women with both lymphoedema, and mastectomy surgical effects being reported as physical movement restrictions.

#### Enablers

Few women spoke of enablers for physical capability, except for one woman who described a benefit from being given specific exercises from an exercise physiologist.

### Capability – psychological

#### Barriers

Some women described difficulty in self-regulating food or drink intake for reasons such as “*stress, defiance but mostly enjoyment*” (ID 11). They had difficulty regulating food intake in response to stress or physical illness, and this could be a result of a complex interplay of physical and mental processes, or due to a non-cancer reason such as work stress. One woman simply described one of her barriers as “*Self-sabotage!!”* (ID 74).

*“Coping with physical changes has been difficult, and coping with the after effects of cancer has left me troubled (e.g. not being able to have children). Rightly or wrongly, I overindulge in food and alcohol, with no reason to stop” (ID 259).*

Lack of support and specific information from healthcare professionals was another barrier to maintaining a healthy weight. Women described receiving conflicting advice from healthcare professionals, a failure to validate their concerns about weight gain, and vague, non-specific advice that was unhelpful.

*“I just get a telling off whenever I go to my oncologist who simply suggests more exercise” (ID 8)*

*“My oncologist and breast care nurse never saw my weight gain as an issue (even when I raised my concerns). I felt discouraged by my treatment team, weight gain seemed a normal part of treatment and life after”( ID 62)*

#### Enablers

Staying positive, creating good habits and being able to access specific programs or support from trained healthcare professionals (including exercise physiologists, general practitioners [GPs], nutritionists and psychologists) increased women’s capability to manage their weight through diet and exercise. Women described using the skills of self-regulation and self-monitoring (often referred to as “*willpower*” and “*accountability*”) and having a clear goal as enablers of weight loss and weight maintenance. As a result, this increased their confidence in being able to manage their weight. One woman described being able to move on from being a breast cancer survivor and being with “*average people*” as an enabler as it meant “*I’m not treated as special. Just normal part of society!*” and it had gotten her out of the “*poor me, why me?*” mindset (ID 219).

### Opportunity—physical

#### Barriers

A range of environmental barriers were described which limited the time and energy available to maintain a healthy lifestyle. These included study, work and family commitments, or being in hot climates. Another barrier was not being able to afford services and programs that could help with weight maintenance, especially in the context of the financial burden imposed by having cancer.

*“The cost of support programs adds additional financial strain; if there was a subsidised scheme for breast cancer patients it would be easier to tackle the issue and set realistic, achievable goals” (ID 19)*

#### Enablers

Access to affordable programs, keeping high-calorie foods out of the house, and having a dog to walk to encourage physical activity were some of the enablers of diet and exercise.

### Opportunity – social

#### Barriers

Women described how the social impact of undergoing cancer treatment impacted on their ability to manage their diet. Friends and family “*made meals for me that I would not normally eat”* (ID 124) and there was an increase in social eating both during and after treatment. Some women also described their friends, family and healthcare professionals discouraging weight loss or not taking their weight gain seriously.

#### Enablers

Support from broad community networks appeared important, whether this involved providing and encouragement or motivating women to take part in group activities. Peer support, whether from people with cancer or people without, was a strong enabler of being able to maintain healthy lifestyle habits.

*“*There was also a Facebook private group where we could post our diets, what we were eating and get support with the other people taking part in the transformation. *The support is wonderful” (ID 219)*

### Motivation – automatic

#### Barriers

Women described a “*loss of willpower”* and turning to comfort eating as a result of feeling depressed or weak following their treatment, while others described disliking exercise. Others identified fear of cancer recurrence as a barrier to weight maintenance.

*“I think I have a sub conscience [sic] fear of weight loss as it has always meant the return of cancer or someone unwell, so as soon as I lose weight I put it back on by eating more! I realise this is irrational but it seems to happen every time!” (ID 100)*

#### Enablers

Few women described enablers with regard to automatic motivation, with the exception of one woman who said she did not “*like the feeling of my thighs rubbing so will lose weight until that happens*” (ID 285).

### Motivation – reflective

#### Barriers

Futility and frustration around weight management was a commonly reported barrier with several women believing there was little they could do about their weight loss with one woman stating that ‘*cancer patients get fat and stay fat’.* Others described a love of food and cooking as a barrier to healthy eating, with one woman saying “*life was too short”* to be eating “*hardly anything”* to prevent weight gain (ID 109).

*“I try programs but after a month of trying hard and not losing weight give up in frustration! Nothing has worked. Need to lose at least 5 kgs but won’t budge. I get despondent” (ID 201)*

#### Enablers

Some women had managed to embrace healthier lifestyles, motivated by the end of their treatment or even the experience of getting cancer itself. One woman described cancer as her “*wake-up call”* to focus on herself and reported being the healthiest she had ever been (ID 158). Other women were clearly motivated by the physical and psychological benefits of being active believing these activities were integral to prevention of mental health and breast cancer treatment-related physical issues. Women were motivated by wanting to get fitter, look better and feel better, and avoiding cancer recurrence. A focus on healthy eating to prevent recurrence was identified as an enabler of motivation, as well as being told to lose weight by a trusted healthcare professional. Adopting healthier lifestyles was seen as a form of self-care and transformation after cancer. Others wanted to know the cause of weight gain, and one woman noted that the fun and welcoming environment of her gym along with the financial and other incentives offered for achieving goals, was a strong motivator to maintain a healthy lifestyle.

*“At the end of the 28 days there was an award for the person that conquered the most demons on their journey and 2 scholarship memberships on cheaper rate. And I won one!... There’s a notice board with positive affirmations, there are little Buddhism sayings on the walls, relaxing pictures, the music is great, the instructors have positive and happy energy” (ID 219)*

### Intervention functions

**Fig. 1:**
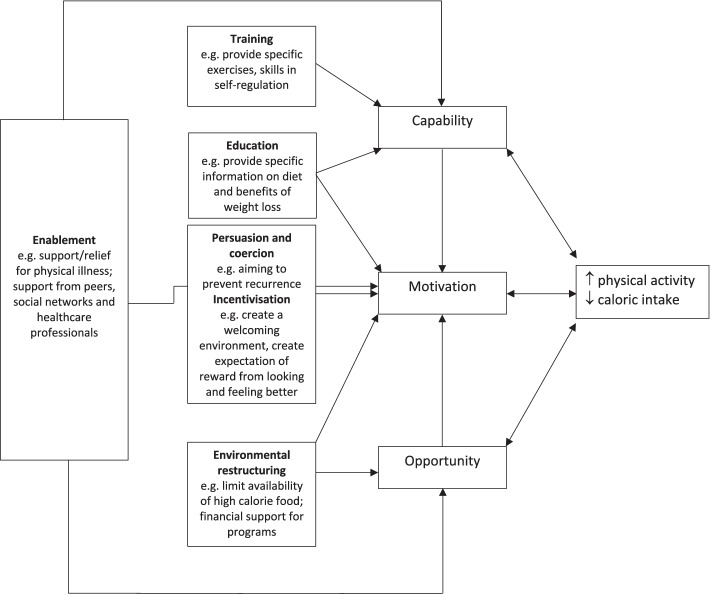
Mapping of intervention functions to COM-B components

Figure 1 depicts the mapping of the intervention functions identified by Michie *et. al.* according to the COM-B components identified in our study. In order to maximise capability, women require training (attaining skills) how to self-regulate, set goals, and self-monitor their behaviour and education (provision of specific information on what exercises are beneficial and what foods they should eat). Maximising opportunity to perform weight loss behaviours requires environmental restructuring (including the provision of additional financial support, important in the context of the financial toxicity of cancer). Persuasion, coercion and incentivisation can be used to create a sense of reward from looking and feeling better, and motivate women to change behaviour with the aim of preventing recurrence of cancer. Last, enablement (beyond environmental restructuring, training and education) is required to optimise all components. This includes efforts to relieve physical symptoms, and provision of support from peers, social networks and healthcare professionals.

## Discussion

In this theory-based thematic analysis of free text responses to a national survey, we identified barriers and enablers of weight management after breast cancer and mapped these to intervention functions using a rigorous and comprehensive theoretical model as a framework. Ours is the first study to apply a COM-B based analysis of weight loss behaviors in women with breast cancer. These findings provide an important foundation to underpin behaviour change interventions to assist women in preventing weight gain and/or achieving weight loss after breast cancer treatment.

In women with breast cancer, weight loss interventions combining diet and exercise interventions with behaviour modification have generally been shown to achieve modest weight losses [7, 15]. A recent Cochrane review reported that multimodal interventions appeared superior to interventions offering diet interventions alone (MD -2.88 kg, 95% CI -3.98,1.77 kg) for change in body weight and concluded that further research is required to determine optimal weight loss interventions for women with breast cancer [7]. Given the importance of providing women with behavioural support to achieve sufficient weight loss together with lifestyle interventions, our findings make a significant contribution to informing the development of optimal behaviour change interventions.

Previous qualitative research has reported several barriers to weight loss after breast cancer, including physical (e.g., the ageing process), environmental/organizational (e.g. traditional female caregiver roles), and psychosocial barriers (e.g. dislike of the gym) [16, 17]. Similarly, we have previously reported on barriers and facilitators of weight management from our national survey of women living with BC in Australia [8]. We identified the most common barriers to successful weight management as being lack of motivation, fatigue, and difficulty maintaining weight, consistent with findings from a recent scoping review [18]. However, the current study is the first to systematically map these barriers and enablers using the COM-B theoretical model. This has practical significance as it facilitates structured mapping of these barriers and enablers to specific behaviour change strategies and techniques, based on the intervention functions identified, which we discuss below.

### Enablement

A key intervention function identified through our analysis is enablement. Enablement of a behaviour refers to increasing the means to and/or reducing barriers to increasing capability (beyond education and training) or opportunity (beyond environmental restructuring). Enablement of weight loss behaviours in women with breast cancer requires a comprehensive approach incorporating supportive care, management of comorbidities, and support from peers, social networks, and healthcare professionals. We found the impact of physical illness, both cancer and non-cancer related, on women’s ability to undertake weight loss behaviours was profound. These findings are consistent with a qualitative study of 17 women with breast cancer, the undesirable effects of cancer treatment on diet as well as physical activity were noted by participants [19], including fatigue, and dietary changes due to chemotherapy effects. In particular, many women attributed weight gain with use of hormonal therapies such as tamoxifen. Although randomised controlled trials have not reported differences in weight with tamoxifen compared to placebo [20] or between anastrozole and tamoxifen [21], younger women were found to be more at risk of weight gain, and these trials only included postmenopausal women [22]. It is plausible that tamoxifen may further exacerbate body composition changes that occur at the onset of menopause [23]. Women on hormonal therapy, particularly tamoxifen, should be provided additional support for weight gain prevention and weight loss.

Our findings highlight a gap in provision of adequate whole-person supportive care. Symptoms such as fatigue, pain, cognitive deficits and anxiety are common among cancer survivors and may present for years after treatment [24, 25]. Women with breast cancer experience a number of psychological symptoms during the cancer continuum including anxiety, distress, depression, and body image dysfunction [26]. Cancer survivors report not being prepared of the health risks of the post-treatment phase [27]. Oncology-led survivorship care is not sustainable due to increasing numbers of survivors [27]. There is growing recognition that cancer survivorship must shift towards a chronic disease model, with primary care needing to play an increasingly larger role due to the significant burden on hospital-based care [24, 28]. Randomised controlled trials have already demonstrated the safety and effectiveness of shared follow-up care [29]. GPs are ideally placed to provide high-quality whole person care for survivors by providing lifestyle advice, assisting with symptoms management, comorbidities and psychosocial issues, as well as referral to multidisciplinary teams (e.g. dieticians, exercise physiologists, psychologists) and community-based programs [24].

Fatigue is common after cancer [30], with other studies reporting this as a common barrier to physical activity in BC survivors [31]. We have also demonstrated lower physical activity levels in women who cited fatigue as a barrier to weight management [9], yet one of the most effective treatments for post-cancer fatigue is exercise [32, 33]. Prescribing exercise to people with cancer can improve cancer-related fatigue, quality of life and physical function [34]. Exercise training was recommended by the 2018 American College of Sports Medicine Roundtable as a means to improve common cancer-related health outcomes including anxiety, depressive symptoms and fatigue [35]. This underscores the need for enablement of these women to make sustainable changes to their physical activity, through alleviation of their physical symptoms, and also training required in altering physical activity according to capability. Our findings provide further validation for the benefits of exercise prescription after cancer.

Further, healthcare professionals caring for women with cancer should acknowledge concerns about weight gain and be able to offer meaningful advice on how to approach weight management. We found the experience of weight gain in itself is a stressor, as have others. In one qualitative study, women with breast cancer expressed surprise and concern associated with changes to weight and diet [19]. In another study of African-American women with breast cancer, participants reported any change in weight (gain or loss) as a stressor that caused psychological distress and health concerns, with frustration at lack of control [36]. Other women may require support to address fear of recurrence, and therefore fear of weight loss, which was identified as a barrier. This finding is consistent with previous research which has highlighted that both weight gain and weight loss may trigger fear of recurrence [37]. Support, acknowledgement and timely referral by healthcare professionals is key in enabling women to optimize their weight after breast cancer, especially in the context of managing psychosocial stressors and existential threats.

We have previously reported that informal social support was cited as the fourth most important enabler of successful weight management in women with breast cancer [9]. Similarly, other studies describe support and positive family and social environments as a facilitator of weight loss in breast cancer survivors [38]. A study of Turkish women with breast cancer reported that women identified a number of needs including that their spouse and family needed to also receive information on healthy living [39] and the importance of household members in dietary decision making was noted in another study [40]. Efforts to raise awareness about the importance of healthy lifestyle habits after breast cancer should involve the woman’s family and other support networks, to provide a supportive environment for the woman and increase chances of successful behaviour change. This is especially important in the context of the change in eating habits occurring with an increase in social encounters and provision of meals by others after a cancer diagnosis, described by some women in our study.

There is mounting evidence for peer support models in supporting healthy lifestyle behaviour change and maintenance for weight loss and disease management, particularly diabetes [41–44]. Peer support is support received from someone with similar characteristics to a patient and experiential knowledge changing lifestyle behaviours. Although few peer support interventions specifically for women after breast cancer have been evaluated, evidence for this type of support for lifestyle behaviour change in general, suggest it warrants future investigation.

### Environmental restructuring

In our study, women described a range of external competing priorities leading to a sense of overwhelm and inadequacy. Similarly, Befort et al*.* reported psychosocial factors associated with weight gain after breast cancer included relationship changes and financial stressors [45]. A qualitative study of women with breast cancer who took part in a diet and exercise intervention found the logistics of fitting the lifestyle program into work and family was challenging [46]. Women in our study also described the financial burden of having to pay for additional programs and support, further exacerbated by the financial toxicity of cancer [47]. This further underpins the need for both holistic physical and psychosocial care among women with breast cancer with implementation of strategies such as financial support and advice as part of environmental restructuring to facilitate behaviour change.

### Education and training

We also identified an unmet need for some women to gain skills in self-regulation. A number of women had already mastered skills such as goal setting, self-regulation and monitoring, and creating good lifestyle habits. Others were unable to regulate their eating in response to stressors, and described a disinterest from healthcare professionals when they expressed concern about weight gain. This highlights the need for behavioural support to equip these women with the skills necessary to help them achieve their lifestyle and weight goals.

### Persuasion, coercion and incentivization

Many women in our study spoke about being motivated by looking and feeling better with physical activity, and by the desire to avoid recurrence of cancer. This is supported by evidence that physical activity alleviates depressive symptoms in women with breast cancer [48]. Physical activity has also been found to be seen as a way to regain control and reduce distress after breast cancer [49]. A review found the experience of physical activity was positive among women with breast cancer with multiple benefits, including empowerment and a sense of reclaiming health [50], consistent with findings from our study. To optimize this motivation, health professionals should encourage women with breast cancer to adopt healthy lifestyle habits both for weight management as well as additional benefits to mental health and prevention of recurrence.

### Strengths and limitations

This paper has several strengths. The nature of the open-ended questions facilitated a broad range of responses, with the large sample size (133 women and 250 responses) allowing us to be confident we had collected comprehensive information. We employed strategies to ensure trustworthiness to ensure coding of data was conducted with rigor. The use of a well-established and comprehensive theoretical model adds further rigor and impact to our analysis and findings. However, we acknowledge that in-depth interviews are more likely to yield further depth in responses and that our findings should be triangulated with data from other sources. Additionally, the survey was only offered in English so non-English speaking participants were excluded. Similarly, the sample lacked cultural diversity, with 94% of women of Caucasian background, which is a further limitation of our study. Our sample consisted of mostly highly educated women. Therefore, we were unable to explore the barriers and enablers of weight management in other subgroups of women such as those experiencing financial distress and those from minority and non-English speaking populations. This is of importance as people from culturally and linguistically diverse backgrounds have significant unmet needs after cancer diagnosis and treatment [51]. Our study findings should be interpreted with this in mind. We did not specify weight loss behaviours (e.g., diet, exercise) when we asked women about barriers and enablers, and therefore it was challenging to interpret some of the free text responses.

## Conclusions

Our theory-based analysis identified what needs to shift in order for women with breast cancer to perform weight loss behaviours of restricting caloric intake and increasing physical activity. Enablement via provision of support from peers, social networks and healthcare professionals, and comprehensive supportive care, impacts on all key components of behaviour change. When combined with environmental restructuring, incentivization (creating an expectation of looking and feeling better), persuasion and coercion (aiming to prevent recurrence), and equipping women with specific knowledge and skills, these intervention functions will expand women’s capability, opportunity and motivation to undertake the lifestyle behaviours that can lead to optimal weight management after breast cancer. Healthcare professionals play a key role in assisting women with weight management after breast cancer and should aim to provide a comprehensive approach to enablement in order to help women meet their weight management and wellbeing goals.

## Supplementary Information

**Additional file 1:** **Table 1.** Additional Quotes - Capability, Physical. **Table 2.** Additional Quotes - Capability, Psychological. **Table 3.** Additional Quotes - Opportunity, Physical. **Table 4.** Additional Quotes - Opportunity, Social. **Table 5.** Additional Quotes - Motivation, Automatic. **Table 6.** Additional Quotes - Motivation, Reflective.

## Data Availability

The datasets used and/or analysed during the current study are available from the corresponding author on reasonable request.
