# Supplementary material for: Barriers and enablers of weight management after breast cancer: a thematic analysis of free text survey responses using the COM-B model
Source: BMC Public Health. 2022 Aug 20;22:1587. doi: 10.1186/s12889-022-13980-6 (PMC9392910; doi:10.1186/s12889-022-13980-6)
Supplement: Supplementary file 1 — Additional file 1: Table 1. Additional Quotes - Capability, Physical. Table 2. Additional Quotes - Capability, Psychological. Table 3. Additional Quotes - Opportunity, Physical. Table 4. Additional Quotes - Opportunity, Social. Table 5. Additional Quotes - Motivation, Automatic. Table 6. Additional Quotes - Motivation, Reflective. [file 12889_2022_13980_MOESM1_ESM.docx]

**Additional Table 1: Additional Quotes - Capability, Physical**

| **ID number** | **Excerpt** |
| --- | --- |
| **BARRIERS** | |
| *Diet: Limited food options due to health conditions* | |
| 103 | I am coeliac and so it is harder to have a filling, high-fibre diet |
| *Diet: Symptoms from treatment affects eating habits* | |
| 124 | Now that I am feeling stronger I am looking to get back to healthier regular eating |
| 127 | Nausea (a general reaction to tiredness, headache, and due to continuing chemo); aversions to sight of foods (eg red meat); smell of food cooking -- once I've cooked it, I don't want to eat it, so opt for faster alternative (or cook in advance and reheat quickly) |
| *Exercise: Physical illness makes exercise difficult* | |
| 3 | I broke my leg & hip in a car accident 2 years ago and still have ongoing issues with this, combined with the lymphoedema in my arm & issues with my breast reconstruction (capsular contracture caused by radiotherapy) it is very difficult to exercise properly |
| 5 | Peripheral neuropathy and feet problems prevent me from walking for longer than 10 minutes at a reasonable pace without ending up in a immobilization boot. Hashimotos [sic] thyroiditis has caused major fatigue |
| 9 | At my age, 75, I find that my mobility is reduced due to circulation in the lower limbs and I am now having other health issues and my balance is a little unstable. I am concerned about having a fall |
| 10 | I am not very comfortable without prosthesis in group/class situations - and exercising with prosthesis is hot and problematic! |
| 12 | I suffer from extreme fatigue |
| 49 | Have lymphoedema in right leg due to melanoma. At times restricts what exercise I can or can't do. Very frustrating. Wrist surgery which has stopped me going to the gym |
| 54 | MS affects exercise both as use 4 wheel walker most of the time, heat sensitive and to some degree fatigue all contribute as well as intermittent steroid treatment |
| 58 | developed rheumatoid arthritis after hormone therapy and now take methotrexate |
| 95 | Osteo arthritis exacerbated by taking Femara; significant knee damage and joint pain means that I cannot undertake weight-bearing exercise anything like what I did prior to diagnosis/treatment. |
| 114 | Arthritis affects ability to walk long distances |
| 119 | Osteoarthritis (hips), meniscus tears (both knees) and moderate obstructive lung disease (from radiotherapy). |
| 125 | neuropathy and left axilla /mastectomy side effects affect some physical movements |
| 137 | I am unable to exercise due to injuries sustained in a motor vehicle accident. |
| 153 | I have experienced very painful feet and joints since I finished chemotherapy and this remains to this day. As a result I have reduced my exercise considerably which then caused weight loss, which then aggravated my arthritis in knees and ankles (as a result of a car accident when I was 18). I know that I would feel better and lose weight if I exercised but it is incredibly painful and therefore something I actually avoid. |
| 164 | I put on 15 kilos with steroids and chemo. Heart got damaged and fluid overload persisted. Have had angioplasties, but still get short of breath on exertion so unable to walk more than 250 metres without rest. Have reactive arthritis and a clash in my essential medications has the side effect of pain in my leg bones with every step. Also had bilateral pulmonary embolisms and dvt on chemo so walking and breathing very restricted. Still improving 2 years later, but it is slow. I have had to accept that this is the new me and I do daily exercises of the arms and legs to maintain muscle tone and strength. I used to walk a lot and now I cannot. |
| 288 | I have back problems which make movement difficult and painful. I used to like to walk but now this is difficult. |
| 301 | Osteoarthritis much worse after Aromasin |
| *Both/unclear: Menopause, physical illness and endocrine therapy make weight loss difficult* | |
| 105 | I think being post-menopausal had something to do with it |
| 109 | Back problems - stenosis; I have found it impossible to lose weight since using tamoxifen |
| 135 | Put weight on during Femara treatment...I was always not overweight or even underweight before, lost weight on the active up-front treatment, but gained it all back and then some on the Femara treatment, especially around my waist. Even 6 months after stopping it has still not gone, despite limiting food & reasonable amounts of exercise. This is not well noted as a side effect |
| 158 | Tamoxifen does slow down your metabolism. I don't care what the GP says..It does! Especially when combined with Cipramil! I eat so much less than I did before diagnosis yet have gained weight. admittedly I have stabilised at 93kg for the past three years |
| 198 | If I had known that the hormone treatments would put on weight I would have been able to control my weight before it got out of hand |
| 256 | I hate everything about being on tamoxifen. It has lead [sic] to weight gain & overall joint pain & bloating. I lift weights, see a personal trainer once a week, walk daily, do yoga & run regularly. I have a good diet & don't eat junk or processed foods. Yet nothing will shift the weight I've gained since starting medication |
| 316 | I am told by my peers that Tamoxifen and menopause in general cause weight gain |
| 317 | I am on Tamoxifen and Zoladex which has suppressed by estrogen levels which I believe has affected my metabolism and weight gain |
| **ENABLERS** | |
| *Exercise: Given specific exercises to use by a trained professional* | |
| 164 | My experience has been to follow specialists [sic] advice. Good diet and exercise programs readily available if you can get a medical clearance to do them. The exercise physiologist and cardiac rehab program gave me the exercises I use |

**Additional Table 2: Additional Quotes - Capability, Psychological**

| **ID number** | **Excerpt** |
| --- | --- |
| **BARRIERS** | |
| *Diet: Lack of interest/vague advice from health professionals* | |
| 62 | differing opinions of professionals. ..... My G.P. advised against changing my diet (as she feels no reliable evidence exists regarding best diet post cancer treatment) |
| 235 | Acknowledgement from health professionals would be good. I felt I should reduce my weight and wanted to do so partly because I saw it as a way of reducing my cancer risk. Not being able to reduce weight then became something I was worried about. It was hard work to lose it but very easy to replace it plus |
| 237 | As long as you don’t have cancer again though, docs and oncs aren’t really interested in such topics, in my experience |
| 256 | the Drs attitude is if it's only 4kg you've gained then you're doing well!!! There is no sympathy or understanding that you've already had your body permanently disfigured because of the cancer but to have your self confidence knocked with weight gain is completely demoralising. |
| *Both/unclear: Distress* | |
| 167 | 5N2 worked...then I was restructured from my job into redeployment and although I have contracts and a great job i have not regained my permanancy and i find that distressing it my age of 55. So stress plus post cancer fatigue contribute equally in disrupting my life |
| *Both/unclear: Lack of information* | |
| 8 | I have put on a considerable amount of weight since treatment for BC. Support in this area is lacking. I just get a telling off whenever I go to my Oncologist who simply suggests more exercise. It would be great if there was some research specific to this issue and suggested programmes to follow |
| 8 | I think I would try anything if there was research to back it up |
| 62 | Lack of knowledge, lack of available information, differing opinions of professionals. Over 3 years I tried many popular diets and exercise programs and nothing worked to reduce the weight.part of treatment and life after |
| 204 | I am a long term survivor and had to sort my way through treatments and what was available at the time I think that there is much more support and information available now - thank goodness. I found practitioners at the time I was diagnosed just concentrated on trying to get me as a patient to survive |
| 293 | Overall I struggled a great deal after having cancer bc I gained 15 kg during and after chemotherapy. I felt depressed had low energy and felt I had no willpower. My oncologist kept telling me to just eat less |
| **ENABLERS** | |
| *Diet: Self-regulation* | |
| 82 | cutting back on amount eaten |
| 105 | I consulted a dietician who helped a little, rejoined Weight Watchers which helped a bit more, but the thing that helped me successfully lose the weight I wanted to lose and keep it off was intermittent fasting. |
| 179 | Self-determination and reducing my intake...I have not had any significant problems with weight gain or loss related to my cancer diagnosis. I have always monitored my weight and been conscious about making sure that I do not gain significant amounts of weight and that I keep my weight within a relatively healthy range, mainly by using dietary intake manipulation |
| 194 | 1. keep a regular exercise program that I can maintain 2. self control on the amount and the type of food taken ie small amount and lots of fruit and vegetables 3. Stay positive |
| 269 | WW as you just eat normally and can eat anything so long as you account for it |
| 295 | The old adage of eat almost anything you like in moderation applies to breast cancer patients as much as it does to anyone else in the community |
| 82 | cutting back on amount eaten |
| *Diet: Specific information about diet* | |
| 61 | I was identified as having a fructose intolerance which made me decrease my sugar intake immediately. This had cause me severe stomach pains. So that was good motivation to lower my sugar intake and has helped me lose a few kilograms. ...Information re lowering sugar intake |
| 165 | Education re good foods and good quantities |
| 219 | There was diet support, recommendations (how to go into ketosis, foods to eat etc), It was run by an exercise physiologist and other gym instructors … attend a gym set up with dietary support, accountability, supervision, |
| 287 | Cooking for myself and increasing the amount of raw food, particularly vegetables, in my diet. |
| 62 | My own research on diet - whole foods, low sugar, no dairy, avoid soy and processed foods, fasting. After much research I reduced my sugar intake, stopped my small intake of dairy, starting consuming whole vegetarian foods and avoiding processed foods and soy. As a result I lost 10kg, reduced my level of daily fatigue and have been able to be more active. Overall my quality of life has improved as a result of diet. I also fast once every six months |
| *Exercise: Creating good habits* | |
| 194 | I keep a regular exercise program that I can maintain. |
| 296 | I have made gym attendance (classes and independent workouts) a regular part of my week. |
| *Both/unclear: Specific program and support* | |
| 5 | Weight watchers. I lost 12kgs and then had thyroid flare up and put 10 back on with no change in my diet |
| 34 | A naturopath helped during treatment and afterwards |
| 51 | Regular support from a Dietician or my doctor has helped in the past Finding a eating approach that works with my altered metabolism would be great |
| 157 | After nearly 10 years & lots of exercises, I have just recently been able to get my weight back into check with the Isagenix program. I have lost 8 ks & would like to loose another 2 ks. For 8 years I have done dragon boat paddling three times per week which has not only prevented me having issues with lymphoma but, has given me a happy mind, body & spirit & a good fitness level. I also attend a fitness group one day per week & the Gym for strength training one day per week. |
| 159 | Nutritionist/dieticians have helped through GP care plan |
| 167 | 5 n2 worked for me 3 years ago and i got down to 83 kg from 88. |
| 247 | Nutritionist |
| 288 | I managed to lose 8 kg with Slimming World in about 3 months but weight loss has been static in last four months. |
| 259 | Psychological support, and perhaps discounted access to an Exercise Physiologist or Personal Trainer. |
| 286 | I attended a rehab program after finishing treatment. It was a combination of excercise and psychological support. |
| 8 | It would be great if there was some research specific to this issue and suggested programmes to follow. |
| 31 | Nutritionist. And Pilates instructor |
| *Both/unclear: Having a clear goal* | |
| 4 | My plastic surgeon told me I needed to lose weight ahead of my breast reconstruction. I lost about 12 kg leading up to the surgery. That was about 6 years ago and the weight has piled on since then. At that time I had a very clear goal and a person I respected keeping me motivated to achieve that goal. |
| *Both/unclear: Psychological support, positive mindset* | |
| 194 | Stay positive |
| 219 | Yin yoga is a lot about letting go and this aspect of the gym where I attend is what has helped the most I think. Letting go of the crap, emotions, sadness, past losses, trauma has really helped me move through and on from cancer and adjust to the new normal. I continue to see a psychologist fortnightly. A cancer diagnosis is usually attached to so much more - to other traumas in your life etc. treatment for weight, exercise needs to be wholistic |
| *Both/unclear: Self-regulation and monitoring* | |
| 101 | My weight has not changed at all. I manage my weight thru exercise and what I put in my mouth. I feel I am lucky when it comes to this as i realise I am in the minority. |
| 41 | Self discipline and willpower |
| 68 | Will power and discipline |
| 131 | Dietician Logging all foods I eat and all exercise. |
| 191 | I'm accountable to myself. I also had support from Curves gym staff. |
| 200 | willpower |
| 241 | Willpower |
| 290 | Mostly self-motivation, reading and learning about maintaining a healthy weight, eating right, exercising and following the 5-2 diet straight away when i've put on a few kilos and my clothes start to get tight!!!... |
| 308 | My own self discipline mind set. Personal accountability. Recording of progress. |
| 312 | A sensible diet, exercise, and self control. |
| 320 | Being accountable to myself |
| *Both/unclear: Self-efficacy* | |
| 220 | Strength of character. I am so tired of people making excuses when they are just weak and need to be sensible about diet and exercise. It's not rocket science. We are responsible for what we eat and how we exercise. If I couldn't do something I normally could because of my surgery, I found other ways around it. The only problem was losing about 5kg after surgery due to stress but my breast cancer surgeon noticed and said I shouldn't lose any more. I am in control..... Making people responsible for their weight. Stop the excuses. |
| 124 | I don't feel that there are any major gaps. It is just a case of doing something about it |

**Additional Table 3: Additional Quotes - Opportunity, Physical**

| **ID number** | | | **Excerpt** |
| --- | --- | --- | --- |
| **BARRIERS** | | | |
| *Exercise: Environment (heat)* | | | |
| 9 | | | Living in the tropics the heat and humidity both have a affect [sic] on my health but I would not be happy moving to another climate. I do swim in my pool and have an exercise plan in the pool |
| *Both/unclear: Lack of time due to study/work/family commitments, general overwhelm* | | | |
| 175 | | | Caring for unwell partner limits time for exercise and cooking |
| 166 | | | There have been medical issues with my husband having 5 aneurysms, requiring two operations to date and another next Monday, over the last couple of months so I guess there has been extra stress and less exercise in some ways but more running around for me in others. However I put on 4 kilos during chemo and extra more recently |
| 110 | | | Moving house, living in temporary rental accommodation |
| 12 | | | I am a single mother of 3 children, two of which are foster children, so time for me to exercise is very limited. I suffer from extreme fatigue, which I believe is a combination of being a single parent of 3 as well as the after affects of my cancer treatment and almost 7 years of Family Court/DV court attendances against my ex-husband, along with other medical conditions. I have had a melanoma and many BCC and SCC skin cancers removed |
| 36 | | | Working 12 hour day shifts now so can only exercise on days off |
| 134 | | | work night shift and am full time carer for husband and was full time carer for father in law until he passed away mid 2017 |
| 158 | | | The weight gain happened quickly and at a time where my mental health and just attending a[appointments had my focus and attention. I also worked throughout my chemo and radiation leaving very little time to exercise outside of work plus deal with the maintenance of my mental health - which to me was vital. |
| 187 | | | Having the time to do adequate exercise. I walk and cycle and find when we are on holidays and have more time/more active weight loss/maintenance is easier |
| 42 | | | Leaving early for work and getting home late each day |
| *Both/unclear: Financial cost* | | | |
| 237 | | | I am on my third medication and weight gain became an issue with letrozole - we will see with Exemestane. I have started working with an ex phys who suggested a dietician but that at this stage that’s another expense |
| **ENABLERS** | | | |
| *Diet: Limiting access to high calorie foods* | | | |
| 226 | | Keeping busy Not having fatty foods in the house | |
| *Both: Affordable programs* | | | |
| 19 | Subsidised financial assistance for weight loss institutions eg: weight watchers, Jenny Craig, dietician services | | |
| 219 | The only thing that helped me was joining a Pilates gym on a 28 day transformation for $197 . The membership is at a price that I can afford | | |

**Additional Table 4: Additional Quotes - Opportunity, Social**

| **ID number** | | | **Excerpt** |
| --- | --- | --- | --- |
| **BARRIERS** | | | |
| *Diet: Other people cooking/social eating* | | | |
| 124 | | | I have done a great deal of social eating while undergoing treatment - coffee and cake and then people have made meals for me that I would not normally eat. I finished treatment (11 months in total) 7 weeks ago and then it has been Xmas etc, so the normal eating challenges |
| 198 | | | Lite n easy where the food is prepared and I don't need to think about food. Problem is going out etc and not following program |
| 213 | | | Hubby serves up large meals, which I eat |
| 285 | | | No barrier as had returned to pretreatment weight by reduced portion size and exercise. However had increased weight after period of travel as special honoured guest at many dinners and lunches. This has now stoped and slow decrease back to normal weight is occurring |
| *Both/unclear: Social pressure not to lose weight* | | | |
| 290 | | | Friends and family tend to think I could go easier on eating and putting on a few kilos won't matter. |
| **ENABLERS** | | | |
| *Exercise: Peer support or support from family/friends* | | | |
| 203 | | My husbands support and encouragement to get back my fitness | |
| 308 | | Holistic approaches. Constant support. Peer group engagement. Societal support for the care burdens of families!! Recognition of unpaid domestic labour within the countries GDP so it is recognised and celebrated as work. Therefore women need to be able to declare sacred exercise time as a non selfish act From other tasks previously deemed “not working” | |
| *Both/unclear: Individualised approach* | | | |
| 119 | Both diet and exercise programs should be designed specifically with and for the patient, not just off the shelf recommendations | | |
| 223 | Identifying the individuals' preferences, and offering the areas that she would prefer | | |

**Additional Table 5: Additional Quotes - Motivation, Automatic**

| **ID number** | **Excerpt** |
| --- | --- |
| **BARRIERS** | |
| *Diet: Eating/drinking for reasons apart from hunger* | |
| 72 | I have to work full time and get very tired so when I am tired I find it very hard to excercise I am on Tamoxifen which put on abdominal fat which us my problem I eat fairly well but do get cravings at times for some sweets or chips and if I am tired I find it harder to resist |
| 124 | Regarding weight, I feel is has been a social/comfort eating . Although not drastic, it is something I want to manage as I feel being leaner will help reduce potential recurrence |
| 287 | I think boredom is one of my main triggers for overeating. And that I genuinely enjoy eating |
| 289 | I LOVE LOLLIES!!!!!!!!!!!!!!!!!! When I give them up the weight falls off but when I am bored at work or have conflicting deadlines, I tend to grab the lollies to power me on |
| 11 | I drink too much alcohol (wine specifically) sometimes due to stress, defiance, but mostly enjoyment |
| *Exercise: Dislikes exercise* | |
| 249 | Hate exercising! Enjoy walking and try to do that daily but then I will have a period where I am busy with housework and get tired and lose interest in walking |

**Additional Table 6: Additional Quotes - Motivation, Reflective**

| **ID number** | | **Excerpt** |
| --- | --- | --- |
| **BARRIERS** | | |
| *Diet: Enjoyment (or dislike) of food and cooking* | | |
| 103 | | And I'm not all that interested in cooking, and my husband doesn't like new dishes! |
| 109 | | I have found it impossible to lose weight since using tamoxifen. Even thought I no longer use it, I still cannot lose weight unless I eat hardly anything and life is too short for that |
| 200 | | I love food and cooking |
| 287 | | I think boredom is one of my main triggers for overeating. And that I genuinely enjoy eating |
| 11 | | I drink too much alcohol (wine specifically) sometimes due to stress, defiance, but mostly enjoyment |
| 249 | | I had a second diagnosis of breast cancer two years ago and found since then I have little enthusiasm for dieting and too much enthusiasm for alcohol. Combined with living with a partner who has Asperger's has meant whilst I know what I need to do, I struggle to do so |
| **ENABLERS** | | |
| *Exercise: Helps mind and body* | | |
| 157 | For 8 years I have done dragon boat paddling three times per week which has not only prevented me having issues with lymphoma but, has given me a happy mind, body & spirit & a good fitness level | |
| 239 | I go dragon boating 2-3 times a week and walk for 30 minutes per day on the other days. I think exercise helps to maintain my weight and assists with combating depression | |
| *Exercise: Look better, feel better* | | |
| 118 | I decided that I was too overweight and my clothes didn’t fit, I felt sluggish and tired and was cross that I was the heaviest I have ever been. | |
| 212 | encouragement from friends and family about how much weight I have lost and how good I look now | |
| 301 | Nothing as I only put on a few kilos around my tummy. I felt uncomfortable as I've never been overweight. I feel better about myself now I have lost it again | |
| *Both/unclear: Knowing the cause of weight gain* | | |
| 198 | If I had known that the hormone treatments would put on weight I would have been able to control my weight before it got out of hand | |
| 267 | Knowing the cause would help greatly. Research on the net has not really helped | |
| *Both/unclear: Wanting to avoid recurrence* | | |
| 112 | I was advised by my oncologist that a weight gain of 5kgs would double my chance of breast cancer recurring. i have been inspired not to put on weight from this comment alone :) | |
| 124 | it is something I want to manage as I feel being leaner will help reduce potential recurrence | |
| 290 | Ensuring people are better informed about the benefits of maintaining a healthy weight, e.g. reduced risk of recurrence. (Easier said than done, I know) | |
| 235 | Acknowledgement from health professionals would be good. I felt I should reduce my weight and wanted to do so partly because I saw it as a way of reducing my cancer risk. Not being able to reduce weight then became something I was worried about. It was hard work to lose it but very easy to replace it plus. | |
| 189 | I think the approach should be more about healthy eating to prevent recurrence etc... than about losing weight. We should be focusing on what we should be doing instead of what we shouldn't be doing | |
| *Both/unclear: Told to lose weight by someone she trusts* | | |
| 4 | My plastic surgeon told me I needed to lose weight ahead of my breast reconstruction. I lost about 12 kg leading up to the surgery. That was about 6 years ago and the weight has piled on since then. At that time I had a very clear goal and a person I respected keeping me motivated to achieve that goal | |
| *Both/unclear: Cancer is a wake-up call* | | |
| 158 | Cancer was a wake up call for me to focus on me. It was a gift | |
